# Anxiety, Boredom, and Burnout Among EFL Teachers: The Mediating Role of Emotion Regulation

**DOI:** 10.3389/fpsyg.2022.842920

**Published:** 2022-03-10

**Authors:** Guorong Shen

**Affiliations:** School of Foreign Languages, Henan University of Technology, Zhengzhou, China

**Keywords:** anxiety, boredom, burnout, EFL teachers, emotion regulation

## Abstract

Teachers’ emotions are explicitly and conceptually presented as part of an educational system that affects and is affected by learner upshots, namely, learners’ self-emotions, behaviors, and cognition since educators and learners are involved in the outcomes of the school setting. English as a foreign language (EFL) educators recurrently experience emotional damages during involvement in their profession as burnout, stress, boredom, and anxiety. EFL teachers need to regulate their emotions when facing a multivariate class environment that provides each learner with undeniable uniqueness. The subject of the relationship between emotion regulation and the teacher’s emotions is receiving increasing attention in research. EFL teachers should be provided with an emotional regulation strategy to have a positive learning-instructing effect in the entire school community as fun learning activities, energetic students, enthusiastic educators, and strong relationships between the board of education. To focus on the role of teachers’ emotion on the one hand and the mediator role of emotional regulation, on the other hand, the current study endeavored to review the role of emotional regulation strategies more intensely to decrease negative emotions. Finally, some educational suggestions of the study regarding the educators’ behaviors are pinpointed.

## Introduction

Emotions have been explored for a long time as an important element of positive psychology (PP) in the field of psychology, but not enough consideration has been drawn to the training of language educators (Dewaele and MacIntyre, 2019; Wang et al., 2021). Emotions have an amazing function in English instruction and education that can lead to more apprehension regarding the EFL/ESL educational setting and the inner associations between EFL/ESL educators and students (Dewaele and MacIntyre, 2019). The possibility of proposing a clear description of this element does not always exist; however, when coping with feelings in the second or foreign language educational cycle, dimensions of the emotion, temper, or demeanor that influence educators’ and students’ dispositions are mainly dealt with (Miri and Pishghadam, 2021). Research in the emotion realm has frequently concentrated on learners’ emotions (Fried et al., 2015). Nevertheless, the “emotional turn” in the language teaching domain has caused interest to the teacher emotion so an increasing number of studies lately has scrutinized teacher emotions, as well (Keller et al., 2014). Relating to this, Schutz and Lee (2014) emphasized the significance of comprehending educators’ feelings to better comprehend language instruction and education.

Educators have gained increasing attention at the center of academia in the last 20 years (Pishghadam et al., 2021). A significant amount of literature emphasizes educators’ attributes as the most important academic characteristic that improves the excellence of education and anticipates the efficient performance of the academic framework (Brown, 2007). Since EFL educators’ beliefs and opinions have great effects on the way they present their profession, they need to have constructive and positive beliefs about themselves, their learners, and the educational cycle and setting (Greenier et al., 2021). Educators encounter a variety of feelings in the class, thanks to communication with their learners, coworkers, managers, and administrators which influence their learners’ presentation and success (Chang, 2013). There are seven distinct emotions educators have in their classrooms such as satisfaction, anger, anxiety, embarrassment, and boredom, and these types of emotions are directly associated with other emotional-motivational paradigms that they may face in the process of their teaching (Frenzel, 2014). Constructive emotions are closely associated with the mental well-being of educators. Regarding this, the utilization of emotions as a means of observing educators’ identity and academic alteration has been highlighted (Van Veen and Lasky, 2005). Previous research indicated that classes are filled more with pleasing emotions than with unpleasing ones. Also, unpleasing emotions are common among instructors (Frenzel et al., 2009); however, negative and unpleasant feelings are correspondingly current among educators (Keller et al., 2014).

Such unwanted but impactful convictions include the EFL educators being in a state of burnout. This is characterized as a mental condition delineated by signals of lessened individual efficacy, emotional exhaustion, and depersonalization due to continuous job inconveniences (Maslach and Leiter, 2016). Burnout may be characterized as static fatigue involving a variety of intellectual, emotional, physical, and societal conditions, all of which emerge from long-run work tension, specifically in professions that require frequent interaction with others (Sulea et al., 2015), Long-run work-related tension, specifically with personnel facility providers and educators, brings about burnout as various researches have demonstrated that educators are exposed to great degrees of tension and burnout, with half of the educators abandoning their careers in the initial five years of instruction (Ingersoll and Smith, 2003).

Another deconstructive emotion that educators might encounter during instruction is anxiety. Educators can be anxious when faced with a lack of readiness to instruct, lack of control over class-related problems, lack of connections with fellow managers and learners’ guardians, and alterations as a result of amendment endeavors (Van Veen and Lasky, 2005). Instructors may experience anxiety in the class, although feelings of anxiety are lower for teachers than for students because teachers do not experience as many obvious failures as students (Frenzel, 2014). Alternatively, boredom is an emotional or mental condition that is common in schools but is ignored and inadequately comprehended by language scholars and educators (Pekrun et al., 2010). Language scholars primarily emphasize anxiety in the L2 class, while educators are inclined to ascribe boredom to an individual’s slack, anxiety, or character elements (Chapman, 2013). Commonly alluded to as one of the curses of contemporary times, boredom is characterized as a deconstructive emotive or mental condition related to an internal feeling of a void in addition to the absence of significance and intention due to a person’s regarding of an educational setting as uninteresting (Goldberg et al., 2011). Bored people regularly encounter a great degree of noninvolvement from their direct educational setting where they take part passively and with indifference. To be more accurate, they have a sense of being estranged from their zeal, wants, or objectives, and find it hard to focus on the present activity because they have no significant engagement (Henry and Thorsen, 2018).

As a whole, the teaching career is full of emotive variability, and attention should be drawn to how this is recognized in the class. That means, to be a dynamic teacher, there is frequently an inherent preparation for the way educators should exhibit their emotional state, and undoubtedly in numerous cases and circumstances, this prospect is more clear and controlled (Greenier et al., 2021). Scholars have been recently focusing more and more on the regulation of educators’ feelings in the class and their impact on educators’ health and learners’ education (Chang, 2013). How individuals manage, oversee, or control their emotional reactions to adapt to existing situations in socially and contextually suitable manners is known as emotional regulation (Pappa and Hökkä, 2021). Indeed, as language educators are under a great deal of pressure due to organizations, peers, and learners, studies on feeling regulation offer educators planned direction on how to deal with deconstructive feelings and difficulties. The control of emotions is crucial for educators to eliminate imminent deconstructive emotions possibly residing in their minds (Myruski et al., 2018). Emotion regulation indicates people’s ability to handle stressful settings and to control their emotions at work (Buruck et al., 2016). Moreover, as stated by Joseph and Newman (2010), people with great capability to adjust emotions are often involved in higher efficient strategies; however, those who adopt suppression strategies show low emotion regulation competence.

In a nutshell, English language instruction is a very emotional career, affected by emotional elements, consisting of both constructive and deconstructive feelings (Nakamura, 2018). Regardless of the interests of these studies, in comparison with other scholastic emotions (for example, exam anxiety and pleasure), there has been relatively a lack of attention to class-relevant boredom, which is one of the most regularly encountered emotions in language education contexts (Pekrun et al., 2010). Certainly, boredom is a deconstructive mental and emotional experience, which could have detrimental repercussions if ignored, including withdrawing from everyday activities as well as suffering from depression (Macklem, 2015). Previous studies of success-related emotions have demonstrated that boredom can cause a variety of discrepancies in school, including, high levels of distraction in the classroom, low internal inspiration, constrained educational engagement, poor educator-learner relationships (Sulea et al., 2015; Lisa et al., 2017). Despite the group of inquiries that have been done so far, to the best of the researcher’s knowledge, there are no review papers that concurrently consider the associations among EFL teachers’ negative emotions such as anxiety, boredom and burnout, and the mediator function of emotion regulation in the language classroom. As an attempt to consider this research gap, the present review scrutinizes the issue in the language classroom.

## Review of the Related Literature

### Emotion Regulation

Emotional regulation is the cycle utilized to regulate feelings, which decides whether, when, and how a person encounters emotional and emotion-relevant inspirational and mental conditions, and how feelings are represented in dispositions (Eisenberg et al., 2014). Emotional regulation or the cycle of regulating the emotions and articulations of institutional objectives or social norms is crucial to comprehending how feelings are encountered and represented during the profession, their influence on workers’ health, and assessments of presentation (Groth et al., 2009; Wang and Guan, 2020). A teacher’s aptitude to manage his own emotional experiences in the context of the classroom is the definition provided for teachers’ emotional regulation. A teachers’ emotional regulation can be conceptualized as the responsibility to the current loads of knowledge with a variety of emotions in a socially acceptable way to allow for a voluntary response and to defer the needed spontaneous response (Cole and Deater-Deckard, 2009). In addition, emotion regulation strategies can be used when instructors try to create caring relationships with students and create an ideal emotional image of the instructor (Sutton, 2010). Reaction-centric techniques and preceding-centric techniques are feeling regulation techniques that educators use afore and following the creation of feelings, respectively (Greenier et al., 2021). Demonstrative subduing and intellectual reassessment are the two primary techniques out of the different reaction-centric and preceding-centric techniques that educators often use to direct their emotions. An effort to recreate emotional occurrences and encounters in ways that change the fundamental significance and effect is known as an intellectual reassessment (Troy et al., 2018). Demonstrative subduing is further characterized as peoples’ attempts to disguise, hinder, or limit feeling-demonstrative manner (Cutuli, 2014).

### Burnout

Maslach and Leiter (2016) argued that burnout is composed of three-dimensional interdependent elements such as emotional exhaustion, depersonalization, and poor individual achievement. When these concepts are extended to educator training, educators experience emotional exhaustion when they are emotionally depleted when associating with others, specifically with their students. A sense of depersonalization occurs when the educator has a deconstructive and unsuitable demeanor toward others, and poor individual achievement is encountered when educators’ expert productiveness and ability are exhausted (Maslach and Leiter, 2016). A person’s sense of being less useful and capable in their profession is known as poor individual effectiveness and it alludes to a deconstructive evaluation of their job presentation and the general worth of their profession (Leiter et al., 2014).

Emotional exhaustion establishes the essential basics of burnout and a person’s sense of emotional void as a result of job stress, disputes, inconveniences, and job overload is alluded to as emotional exhaustion (Skaalvik and Skaalvik, 2010). People may experience exhaustion in these circumstances and may not have sufficient strength and excitement to cope with everyday job difficulties (Fathi et al., 2021). Depersonalization was described as a sense of disinterest and unconcern about one’s work and those to whom one offers service. Fundamentally, people with depersonalization are inclined to regard their work and those they have involved in the workplace in a deconstructive way (Maslach and Leiter, 2016).

The main factors that are thought to contribute to educators building burnout are usually divided into two classes: character and setting. Character factors have been debated in numerous researches and have incorporated character attributes, discerned self-awareness, sense of purpose, productive contemplation, involvement, age, gender, and duration of exercise, willingness to learn, inclination to work, and skills (Baran et al., 2010). Aloe et al. (2014) outlined the results on the duration of practice and the results of classes taught by educators. It was demonstrated that educators of a lower age are inclined to encounter a greater level of burnout compared to those of higher age, in addition to high school educators when contrasted with educators instructing in primary schools. Setting elements involve academic frameworks, the standard of academic organizations, burden, school instruments, time constraint, labor conditions, absence of awareness, legal modifications, social support, connections among peers, learners’ educational issues, large classrooms, etc. (Fiorilli et al., 2015). Educator burnout syndrome could result in absenteeism, which straightforwardly affects learners’ scholastic presentation, results in more penalizations of children, or their indifference (Aloe et al., 2014).

### Boredom

Emotions are considered concepts of several elements, including emotional, mental, intellectual, expressive, and inspirational elements. At the center of emotion is the emotional element and it can be said that emotion is impossible without emotional experience. One definition provided for boredom is a state, emotion, motivation, psychological experience, which is mitigated, unpleasing, or painful, occurring when the environment is boring, monotonous, and unchallenging (Macklem, 2015; Han, 2021; Pawlak et al., 2021). In educational psychology, the control-value theory of academic emotions states that boredom consists of a multidimensional construct (Putwain et al., 2018). Boredom is hypothesized as a destructive and de-activating emotion caused by constant learning activities or assignments, and enjoyment as a positive and activating emotion due to continuous learning activities or assignments (Derakhshan et al., 2021). Anxiety is a negative and activating emotion aroused by an expected outcome about future learning results or performance (Pekrun and Perry, 2014). Keller et al. (2014) investigated boredom among educators and proved that educators encounter considerable boredom from instruction in the class in around one-fourth of all sessions. Boredom is regularly regarded as a psychological condition with less arousal and inactivation. According to the scholars, the outcome needs more research because it contradicts the lively function educators have to employ in the session (Radka, 2021).

### Anxiety

Anxiety is considered a natural human emotion that can be caused by inner or outer alterations, unknown circumstances, or a sense of ambiguity. In other words, it is normal for most people to feel stressed and pressured, which is regarded as anxiety when encountering a specific circumstance that is foreign (Cubukcu, 2007). Likewise, anxiety is also characterized as an unusual and immense feeling of worry and distress, frequently delineated by physiological symptoms, uncertainty regarding the truth and essence of danger, and by self-uncertainty regarding one’s ability to deal with it (Hewitt, 2011). Anxiety is elucidated as a fear or as aimless insomnia after identifying danger, and as a disorder of nervousness and hesitation, definitely about future apprehensions (Yükselir and Harputlu, 2014). Shillingford-Butle et al. (2012) mentioned that teachers come across numerous trials triggering signs of anxiety, including education, law, policies for school reform, guardian-educator relationships, and disputes with other educators. It is held that the condition where educators work obliges them to do badly in their work. For example, considering the physical condition of language education institutions in Iran, most of the time there are ancient buildings with improper ventilation and shabby classes that resents educators.

## Implications and Future Directions

This study makes an effort to arrange for the views of the aspect of teachers’ emotions and associate it with the foreign language teaching milieu. The feelings experienced by the educator are very significant for teaching intentions as they are related to the educator’s manner, the educator-learner connections, and the learners’ presentation (Becker et al., 2014). The literature review reveals the endeavors of arising educator interferences targeting emotion regulation techniques to enhance educators’ skill of dealing with career-related inconveniences and heartening their health (Harris et al., 2016). It can be stated that observing an individual’s passions or emotional regulation is very probable to decrease critical and demanding work practices of educators. Emotional regulation also allows educators to cope with traumatic issues and care for them alongside unfriendly upshots of encountering teaching stress (Myruski et al., 2018). If teaching anxiety and stress are not lessened, educators are very probable to get discouraged, lose their enthusiasm, feel much tired, and have more negative manners toward their learners (Yu et al., 2014), so through emotional regulation, teachers can decrease their anxiety level.

In addition, this review has significant educational implications for not only teacher instruction but also for foreign language education. By encouraging more studies in this domain, pre and in-service educators are taught to change their teaching techniques and contents to negate the negative influences of boredom and select more absorbing ones that will help them conquer boredom and as a result, educators can build a pleasant educational setting (Fathi et al., 2021), which is the basis for engaging students in the educational cycle by regulating their feelings. Educators are advised to enhance their emotional regulation techniques, including constructive feelings that add to problem-solving abilities, following the broaden-and-build hypothesis of positive feelings. Since most of the specified techniques are directed only to feelings, educators must be coached in successful techniques. Thus, this could be one of the course goals of part-time coaching plans and pre-service educator training plans.

Moreover, EFL educators need to have the ability to control their deconstructive emotions sufficiently to promote a more constructive class atmosphere, sustain constructive relationships with students, better control the educational system, and better communicate particular planned issues. Educators, generally and EFL educators specifically need to enhance their instructional abilities, like the regulation of feelings, to avoid transferring such deconstructive conceptions toward their learners. Furthermore, faculty members must offer a suitable instructional setting and carry out workshops to assist educators with disregarding the rise of negative emotions. Involvement in the regulation of emotions can be contended to be very promising in the setting of educators’ study on feelings. It can enhance staff associates’ comprehension of the influences of educator feelings, which is interesting and of fundamental academic appeal. Comprehension of when and how feelings must be regulated by educators is additionally a crucial precondition for developing interferences that aim for educators’ regulation of feelings for enhancing class performance.

It is contended that the inclusion of prearranged teaching in educator training plans has a constructive impact on educators’ emotional abilities, reduces anxiety, and results in long-run enhancement in educators’ professions as Gibbs and Powell (2012) stated, by gaining more dominance over their beliefs and feelings, educators could improve their instruction skills. Teachers who can regulate and manage their feelings effectively are aware of the way to generate or change feelings such as joy and appeal to encourage students to learn (Hen and Sharabi-Nov, 2014). They are conscious of their skills, strengths, and weaknesses in their emotional abilities and they regulate their feelings to maintain constructive associations with their learners, comprehend the feelings of their learners, and come up with answers in hard circumstances. Steps were taken by educators to control, regulate, alleviate, and in some cases, suppress their feelings in the classroom that have a great impact on the quality and scope of their relational connections with learners (Taxer and Gross, 2018).

As a result, the researcher concluded that regulating teachers’ emotion is significant for the teachers as it can decrease the levels of negative emotions and foster positive ones and consequently, educators who encounter constructive feelings in instruction could use a broad range of teaching techniques, are more pliant and imaginative in adjusting to various class circumstances, with concurring favorable influences on learners’ results. Deconstructive feelings, contrastingly, harm the presentation of intricate assignments because they are associated with superficial and little information processing (McKasy, 2020). Educators who encounter a greater constructive influence while teaching learners to announce more career fulfillment and lower levels of burnout. Constructive impacts assist people with fighting deconstructive feelings, elevating health, strengthening flexibility, and developing long-lasting individual assets (Fredrickson, 2001). In addition, it is deduced that teachers can make more suitable choices about classroom incidences when they can adjust their emotions more properly and these choices can inspire them to act better that can also improve their self-efficacy that leads to the ways to decrease or even diminish their burnout and anxiety.

In line with the review of the literature, the role of emotion regulation is significant in dealing with teachers’ burnout as it is a significant element that impedes their competence, productiveness, and professional integration, impacting the standard of education and, as a result, learners’ presentation in a deconstructive way (Azeem, 2010). Indeed, educators with burnout could suffer from a loss of vitality, engagement, and excitement to proceed with their job, and this could leave a deconstructive impact on their learners in the L2 educational cycle. If teachers positively control and regulate the anxiety that may be flowing inside them despite their learners’ misbehavior by the routine of emotional regulation strategies, the negative impacts of their anxiety on their teaching behavior will be lessened, and they can effectively keep with their class, despite the practices of anxiety. In line with this viewpoint, it can be assumed that the emotional practice intrinsically has argumentative properties, which can be alleviated if the passion is positively controlled.

The study provides psychological suggestions for curriculum planners so as not to disregard the function of feelings in the curriculum plan. Tasks and activities that emphasize the function of educators’ feelings must be planned as increasing study in the relevant literature has highlighted the importance of integrating educators’ emotional dimensions into the instructing cycle and the demand to emphasize more educators’ emotional abilities (Hen and Sharabi-Nov, 2014).

It seems that if educator-training instruction classes take into consideration the regulation of feelings as important conflicts in the material of the coaching plans, pre-service educators and trainee educators will possess a greater opportunity to learn more about the way to regulate their feelings, and be capable of determining which elements best affect techniques of the regulation of feelings in the class. Furthermore, for new educators who are beginning instruction, how they enhance their abilities of feeling regulation is highly significant for their instruction and class administration. Teacher educators should be aware of effective emotional regulation and managing strategies to provide teachers with approaches and tactics to comprehend the emotive facets of teaching and assist them not to disregard their emotions. Coping contains classifying and marking what individuals are feeling along with choosing tactics to improve or diminish their feeling (Davis et al., 2008).

The reviews of various studies significantly and timely might contribute to the literature by investigating the role of emotional regulation in the mediation of the negative emotions of instructors. In addition, the researcher beliebves that this review contributes to a broader literature on workplace emotions and more specific literature on language teachers’ burnout, anxiety, and boredom. Finally, the results of this study can also help organizations develop and implement effective instructor support programs and select strategies about the emotions of teachers. More experiential and theoretical research is required, thanks to the dearth of studies on this subject, to investigate the relationship between educators’ feeling regulation and learners’ achievement. Because of the significance of emotion regulation in the scholastic setting, several intercultural types of research have been proposed on the function of this concept and other factors involved with positive psychology.

## Data Availability Statement

The original contributions presented in the study are included in the article/supplementary material, further inquiries can be directed to the corresponding author.

## Author Contributions

The author confirms being the sole contributor of this work and has approved it for publication.

## Funding

The work is supported by Innovation Funds Plan of Henan University of Technology (Grant No. 2021-SKCXTD-13), by Henan Provincial Department of Education - “Henan First-class Post Graduates Course Project -Culture Translation (YJS2021KC13)”, by Henan Provincial Department of Education - “Henan First-class Undergraduates Course Project (Chinese-English Interpreting)” and by Henan Provincial Department of Education (Grant No. 2021YB0099).

## Conflict of Interest

The author declares that the research was conducted in the absence of any commercial or financial relationships that could be construed as a potential conflict of interest.

## Publisher’s Note

All claims expressed in this article are solely those of the authors and do not necessarily represent those of their affiliated organizations, or those of the publisher, the editors and the reviewers. Any product that may be evaluated in this article, or claim that may be made by its manufacturer, is not guaranteed or endorsed by the publisher.
